# Incorporating a Wheeled Vehicle Model in a New Monocular Visual Odometry Algorithm for Dynamic Outdoor Environments

**DOI:** 10.3390/s140916159

**Published:** 2014-09-01

**Authors:** Yanhua Jiang, Guangming Xiong, Huiyan Chen, Dah-Jye Lee

**Affiliations:** 1 Intelligent Vehicle Research Center, Beijing Institute of Technology, 5 South Zhongguancun Street, Haidian District, Beijing 10081, China; E-Mails: price@bit.edu.cn (Y.J.); chen_h_y@263.net (H.C.); 2 Beijing Automotive Technology Center, Beijing 101300, China; 3 Department of Electrical and Computer Engineering, Brigham Young University, Provo, UT 84602, USA; E-Mail: djlee@byu.edu

**Keywords:** monocular visual odometry, motion estimation, pose estimation, vehicle dynamic model, wheeled vehicles

## Abstract

This paper presents a monocular visual odometry algorithm that incorporates a wheeled vehicle model for ground vehicles. The main innovation of this algorithm is to use the single-track bicycle model to interpret the relationship between the yaw rate and side slip angle, which are the two most important parameters that describe the motion of a wheeled vehicle. Additionally, the pitch angle is also considered since the planar-motion hypothesis often fails due to the dynamic characteristics of wheel suspensions and tires in real-world environments. Linearization is used to calculate a closed-form solution of the motion parameters that works as a hypothesis generator in a RAndom SAmple Consensus (RANSAC) scheme to reduce the complexity in solving equations involving trigonometric. All inliers found are used to refine the winner solution through minimizing the reprojection error. Finally, the algorithm is applied to real-time on-board visual localization applications. Its performance is evaluated by comparing against the state-of-the-art monocular visual odometry methods using both synthetic data and publicly available datasets over several kilometers in dynamic outdoor environments.

## 1. Introduction

Self-localization is arguably one of the most challenging problems in intelligent vehicle research. Traditionally, wheel speed encoder is used for auto-localization of wheeled autonomous vehicles. However, the accuracy of localization using photoelectric encoder is oblivious to external environment due to its dependency on proprioceptive sensors. The gross error is inevitable [1] when abnormal situations, such as wheel slippage or sinkage, occur. Another drawback of wheel encoder-based odometry is the non-boundary accumulated error [2]. Recently, a class of vision based localization method has been proposed to address these shortcomings. Using visual information to estimate the motion of a ground robot has been an active field of research in the past 30 years. Monocular, stereo and ominidirectional cameras have all been used in vision based motion estimation systems. A notable successful example of visual odometry is NASA's Mars rovers [3].

Literature review shows that visual odometry (VO) algorithms are divided into feature-based and appearance-based methods. Only feature-based methods are studied in this work because of their efficiency and robustness. As the term suggests, feature-based methods focus on tracking salient and reoccurring features over frames for motion estimation. Based on the principles of visual geometry, the relative camera pose or movement between frames can be estimated from a certain number of corresponding points. Various features, including point, line [4], contour [5], and hybrid [6], all have been used for this purpose. Point feature is the most prevalent of all because of its simplicity. However, because of errors in point feature detection, matching and tracking, data association or outlier removal problem becomes very critical. Feature detection and match algorithm is not the focus of this work. A good overview can be found in [7]. The focus of this paper is motion estimation.

For motion estimation with the presence of outliers, the RAndom SAmple Consensus (RANSAC) paradigm [8] based on hypothesis-and-test mechanism has been established as the standard method. The minimal algorithms mentioned above can act as the hypothesis generator in RANSAC framework. Minimal solvers, such as 5-point RANSAC, for monocular VO, and 3-point [9] RANSAC, for stereo VO, are now widely used [10].

The number of RANSAC iterations increases exponentially as the increase of the number of parameters that describe the motion. Vehicle kinematic constraints have been applied to VO in order to reduce the size of minimal solvers and hence reduces the number of iterations of RANSAC [11–14]. Vatani *et al*. used the Ackermann steering principle and the planar assumption to constraint the motion model [11]. Two-dimensional planar motion was then estimated directly using pixel displacement from a downward-looking camera. Using the vehicle kinematic model, Zhu *et al*. [14] computed the motion parameters by solving a quadratic polynomial from equations established by the epipolar constraint. For general wheeled vehicles, Scaramuzza *et al*. [12,13] showed that, due to the existence of the Instantaneous Center of Rotation, motion can be locally described as planar and circular, and the motion model can be simplified to 1 DoF (Degree of Freedom). This simplification leads to a 1-point minimal solver. Their restrictive model is based on the assumption that the motion is locally planar and circular. This assumption is often violated in outdoor environments even when the road is perfectly flat. If we treat the camera and the vehicle as a spring-mass system, when acceleration, deceleration, or sharp turns occurs, the planar-motion hypothesis will fail due to the dynamic characteristics of wheel suspensions and tires. For these reasons, in their latest work [15], the authors relaxed the locally planar and circular motion constraints. The motion prior to using 1-point algorithm is used to compute the target distribution of the 6 DoF motions, leading to improved performance compared to their 1-point algorithm.

All the aforementioned kinematic model-based methods totally ignore the tire side slip characteristics, which cause deviation in the estimation of translation vector. The effect is evident especially in highway driving or even low-speed steering when the non-linear dynamic characteristics of vehicle are significant. In most practical situations the camera is fixed to sprung mass, which does not satisfy the planar motion hypothesis due to dynamic characteristic of wheel suspension. Hamme [16] also mentioned that the camera orientation with relation to the road plane can not remain fixed due to the compliant suspension system. In this work we consider the change of pitch angle, and make a sophisticated linearization of dynamic model in order to estimate principle motion parameters accurately and efficiently.

The remainder of this paper is organized as follows. In Section 2, we describe the problem formulation and some notations. In Section 3, we introduce both kinematic model and basic dynamic model of wheeled vehicles. In Section 4, we discuss how to involve the vehicle dynamic model in monocular visual pose estimation. In Section 5, we briefly describe the refinement approach. Finally, in Section 6, we present the experimental results on both synthetic and real data.

## 2. Problem Formulation and Notations

Considering a 3D world point *Q* and its projections *q*_1_ and *q*_2_ from two views that are all represented in homogeneous coordinates. A particular 3 × 4 camera matrix *P* can be defined for a view indicating the image projection *q* = *PQ* (up to a scale factor). *P* can be factored into *P* = *K*[*R*∣*t*], where *K* is a calibration matrix holding the camera intrinsic parameters. *R* ∈ SO(3) and *t* ∈ R3 × 1 are the 3 × 3 rotation matrix and 3 × 1 translation vector that represent camera motion between the two views.

The fundamental matrix *F* is defined as:

(1) $$F\equiv K_{2}^{-T}{\left[t\right]}_{\times }RK_{1}^{-1}$$

where [*t*]_×_ denotes the skew symmetric matrix defined by *t*. The co-planarity equation or epipolar constraint can be expressed as:

(2) $$q_{2}^{T}Fq_{1}=0$$

For a calibrated camera, *K*_1_ and *K*_2_ (intrinsic parameters from two views of the same camera) are known and supposed to be the same. The projections *q*_1_ and *q*_2_ represented in image pixels can be converted to normalized vectors projected onto the image plane in the camera coordinate system as *q̂*_1_ and *q̂*_2_. Consequently the epipolar constraint can be rewritten as:

(3) $${\hat{q}}_{2}^{T}E{\hat{q}}_{1}=0$$

where *E* = [*t*]_×_*R* is called the essential matrix, ${\hat{q}}_{1}=K_{1}^{-1}q_{1}$, ${\hat{q}}_{2}=K_{2}^{-1}q_{2}$.

Given five, seven, or eight known point correspondences, *E* can be calculated according to the visual geometry principle. Motion parameters *t* and *R* can then be extracted from *E* using Singular Value Decomposition (SVD). After the camera rotation and translation between consecutive frames are estimated, the location and orientation of the camera (or vehicle) with respect to the vehicle's initial position can be calculated cumulatively.

**Figure 1.**
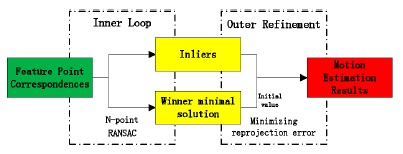
Motion estimation pipeline of two-frame feature based visual odometry algorithm.

In general, vision based motion estimation algorithms consist of two steps: inner identification and outer refinement, as depicted in Figure 1. In the inner loop, feature correspondences are separated into inliers and outliers using a hypothesis-test-validation paradigm, such as the standard RANSAC. The hypothesis generator uses a minimal number of feature correspondences to solve the relative orientation problem. The minimal algorithm is named after the size of the minimal set, typically 5-point [17], 7-point [18], 8-point [19] are widely used. For a calibrated camera, at least five point correspondences are required to determine the relative pose between two frames [20]. Considering common M-point algorithms for solving the relative pose problem, the number of iterations *n*_RANSAC_ that is required to guarantee a stable solution is:

(4) $$n_{\text{RANSAC}}=\frac{\log(1-p)}{\log(1-{\left(1-ɛ\right)}^{s})}$$

where *s* is the size of minimal set, *ε* is the percentage of outliers and *p* is the requested probability of success [8].

Obviously, *n*_RANSAC_ will increase exponentially as *s* increases. A summary of the number of RANSAC iterations needed when s changes can be found in [12].

In the outer step, the winner minimal solution is refined with all the inliers found through minimizing reprojection error or other similar error metrics. The most popular method used as the final refining process is Bundle Adjustment (BA) [21], which optimizes reprojection error over both structure and motion parameters using the Levenberg-Marquardt algorithm.

The main contribution of this work is to incorporate the vehicle dynamic model in the inner loop to effectively select inliers. This approach provides more accurate result than the traditional kinematic model based methods while maintaining the same computation requirements. It demonstrates better performance even with the non-linear dynamic characteristics of vehicle when the vehicle is moving at high speeds. This method is able to handle sophisticated linearization of principle motion parameters and yield promising motion estimation results. This new method is named MYP algorithm for its use of the dynamic Model of Yaw and its consideration of Pitch as well.

**Figure 2.**
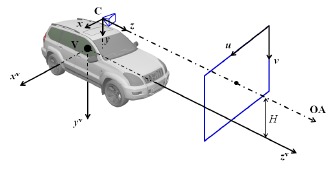
Coordinate system definition.

Respecting the customs in photogrammetry [18], the Camera Coordinate System (CCS) defined in this work is to let *z*-axis point to the forward direction as shown in Figure 2, where V denotes the centroid of the vehicle as well as the origin of the Vehicle Coordinate System while C denotes the origin of CCS; OA represents the Optical Axis of the camera; *u* and *v* are pixel coordinates on the image plane; *H* represents the height of camera mount. The CCS at initial time *t* = 0 is defined as the Global Coordinate System.

## 3. Motion Models of Wheeled Vehicles

In order to reduce the degree of freedom in vision based motion estimation problem, a few researchers have introduced motion models of wheeled vehicle into the pose estimation process. We will present the kinematic model and dynamic model in this section. We will also explain how these models work and the analysis of the model fitness using synthetic and real data.

### 3.1. Nonholonomic Constraints of Ackerman Steering

As mentioned before, the classical Ackerman Steering Principles are used to ensure a smooth motion of vehicle, as well as the camera, where the camera is simply described with local circular motion. According to [12] the rotation matrix *R* and translation vector *t* can be parameterized as:

(5) $$\begin{array}{ll} R=\left[\begin{array}{ccc} \cos\omega & 0 & -\sin\omega \\ 0 & 1 & 0\\ \sin\omega & 0 & \cos\omega \end{array}\right] & t=\rho \left[\begin{array}{c} \sin\beta \\ 0\\ \cos\beta \end{array}\right] \end{array}$$

where *ω* is the yaw angle increment of the vehicle and *β* is the vehicle body side slip angle. The key of 1-point algorithm is the geometric relationship between *ω* and *β, i.e*., *β* = *ω*/2. The essential matrix *E* for the planar circular motion can then be described using a single parameter *ω*, and the closed-form solution of *ω* can be derived. Consider a set of 2D feature correspondences {*^i^u_k_*_-1_↔*^i^u_k_*} and *i* = 1, 2, … *N*, where *N* is the total number of feature points and *k* represents the frame number. For each feature correspondence *^i^u_k_*_-1_↔*^i^u_k_*, the yaw angle increment can be determined using only one correspondence as:

(6) ωi=−2arctanûik−1(2)·ûik(1)−ûik−1(1)·ûik(2)ûik−1(3)·ûik(2)+ûik−1(2)·ûik(3)

where the hat denote the normalized vector of image projection, *^i^û*_*k/k*−1_(*j*) denotes the *j*-th element of vector *^i^û*_*k/k*−1_.

Then, the best estimate of yaw angle increment, denoted as ω*, can be selected through histogram voting or a RANSAC based method. Using ω* and Equation (5), the rotation matrix *R*^1^ and translation vector *t*^1^ can be determined. Here, the superscript 1 represents model estimation results using the 1-point algorithm.

Three model based motion estimation methods, specifically using the 1-point algorithm, are summarized below:

Method 1: Uses {^i^*ω*} and ω* to reject outliers whose estimation error ‖^i^*ω* − ω*‖ is larger than some threshold.
Method 2: Triangulates features with motion baseline *R*^1^ and *t*^1^ to reject outliers by thresholding the reprojection error.
Method 3: Uses stereo triangulated 3D points {*^i^X*} and 2D image projection {*^i^_uk_*_−1_} to reject outliers by thresholding the reprojection error.

Basically, Method 1 can only be used as a coarse filter with lax thresholding. In monocular setting, only Method 2 can work like the original application in [12]. Method 3 works only when stereo vision is available.

### 3.2. Single-Track Bicycle Dynamic Model

As discussed in Section 3.1, the tire side slip characteristics are totally ignored in the kinematic model. The omission of the tire side slip causes deviation in the estimation of translation vector. In [14], the yaw increment *ω* and side slip angle *β* are treated as independent variables which lead to quadratic equations from feature correspondences. We are more interested in the inherent relationship between *ω* and *β*, which is naturally relevant in vehicle dynamics. Single-track bicycle model described by Mitschke in [22] is the simplest dynamic model of wheeled vehicles. This model combines the front and rear wheels and treats the vehicle as a bicycle. It originally describes the vehicle motion in three degrees of freedom (*x-y* position and yaw rate) in integral use.

The following are the differential equations of the bicycle model:

(7) $$\left(k_{\text{F}}+k_{\text{R}}\right)\beta +\frac{1}{v_{\text{long}}}\left(l_{\text{F}}k_{\text{F}}-l_{\text{R}}k_{\text{R}}\right)\omega_{\text{r}}-k_{\text{F}}\delta =m\left({\dot{v}}_{\text{lat}}+v_{\text{long}}\omega_{\text{r}}\right)$$

(8) $$\left(l_{\text{F}}k_{\text{R}}-l_{\text{R}}k_{\text{R}}\right)\beta +\frac{1}{v_{\text{long}}}\left(l_{\text{F}}^{2}k_{\text{F}}+l_{\text{R}}^{2}k_{\text{R}}\right)\omega_{\text{r}}-l_{\text{F}}k_{\text{F}}\delta =I_{y}{\dot{\omega }}_{\text{r}}$$

In these two equations, *k*_F_ and *k*_R_ denote the effective cornering stiffness of front and rear tires respectively and m represents the vehicle curb weight. *I_y_* is the moment of inertia around the *y*-axis (defined in vehicle coordinate system, see Figure 2). *l*_F_ and *l*_R_ are geometric values that denote the distance from the front and rear axles to the centroid of vehicle respectively. *v*_long_ and *v*_lat_ represent longitudinal and lateral velocity and *ω*_r_ denotes yaw rate. *β* denotes the vehicle body side slip angle and *δ* represents the wheel steering angle. There is a difference between *ω*_r_ and pre-defined *ω. ω*_r_ is canonical yaw rate of which dimension is rad/second, while *ω* is yaw angle increment in each interval, its dimension is rad, and their relationship fulfill *ω* = *ω*_r_d*t* where d*t* is the acquisition interval.

### 3.3. Model Fitness Analysis

**Figure 3.**
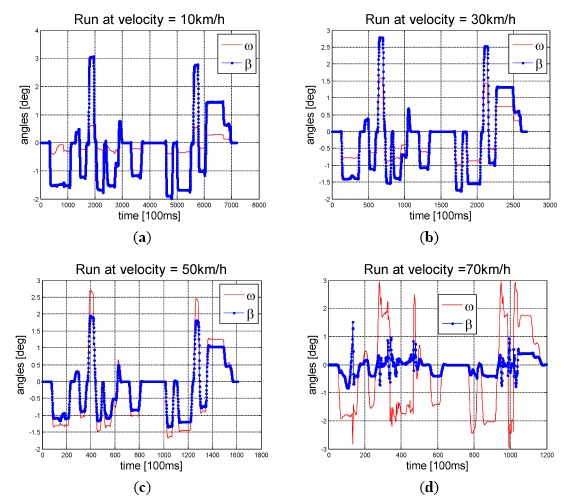
Plots of yaw angle increment *ω* (at 100 ms interval) and side slip angle *β* using CarSim simulated data. Velocity changes from 10 km/h to 70 km/h.

**Figure 4.**
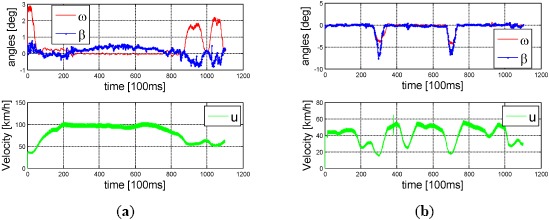
Plots of yaw angle increment *ω* (at approximately 100 ms interval) and side slip angle *β* from the KITTI ground truth data at (**a**) high speed and (**b**) low speed.

We observe the values of *ω* and *β* using both synthetic data and real car data. In Figure 3, we present the plots of *ω* and *β* using CarSim simulated data, and the detail of simulation configuration was proposed in our previous work [23]. It is interesting to note that there is an ideal linear proportion between *ω* and *β* when the vehicle runs at medium or low speed (typically less than 50 km/h). Whereas, when the vehicle is running at high speed, the non-linear relationship becomes noticeable. At the medium and low speed applications, the scale factor between *ω* and *β* varies with the speed. The slower is the speed, the higher is the ratio of *β/ω*. The plots from real car data shown in Figure 4 also confirm this observation.

We compared the angles using one KITTI sequence [24]. The yaw angle increment *ω* and side slip angle *β* for high speed (top left plot) and low speed (top right plot) and their corresponding speed over time are plotted in Figure 4. Both the non-linear proportion of *β/ω* at high speed (60–100 km/h on the left) and the linear proportion of *β/ω* in low-speed steering (around 20 km/h on the right) are far from the *β/ω* = 1/2 assumed in the kinematic model.

**Figure 5.**
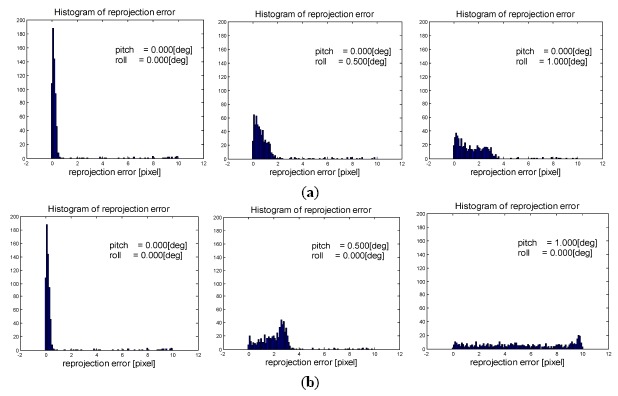
Plots of reprojection error against pitch angle increment and roll angle increment using synthetic data. (**a**) Increase the roll angle increment; (**b**) Increase the pitch angle increment.

We are interested in investigating the influence of model angle bias on the performance of 1-point algorithm. In Figure 5 we give a simple demonstration using synthetic data. The motion is simulated with fixed yaw angle increment while increasing the pitch angle increment and roll angle increment respectively, then the reprojection error are calculated using the canonical pipeline of 1-Point algorithm described in Section 3.1. Figure 5 shows that the increase of pitch angle increment causes more severe damage to the distribution of reprojection error, and finally damages the inlier identification result. Therefore, it is necessary to consider pitch angle increment separately in the model estimation step.

As the inlier identification procedure can be regarded as a binary classification problem, we use sensitivity and specificity as evaluation metrics. Define True Positive (TP) as the number of true inliers found, False Negative (FN) as the number of true inliers missed, True Negative (TN) as the number of true outliers found, and False Positive (FP) as the number of true outliers identified as inliers. To compute the true inliers and true outliers, we use ground truth poses and a reprojection-error threshold of 1 pixel. Then, sensitivity (also called true-positive rate or recall rate) and specificity (or true negative rate) can be computed as:

(9) $$\begin{array}{ll} \text{sensitivity}=\frac{\text{TP}}{\text{TP}+\text{FN}}, & \text{specificity}=\frac{\text{TN}}{\text{TN}+\text{FP}} \end{array}$$

In ideal cases, we would like *sensitivity* = 1 and *specificity* = 1.

The following is an exemplary evaluation of the outlier-removal performance of the original 1-point algorithm [12] and MOBRAS [15] on the publicly-available KITTI dataset. We choose Sequence 05 as it is a very complicated path, with many sharp turns as well as many accelerations and decelerations.

**Figure 6.**
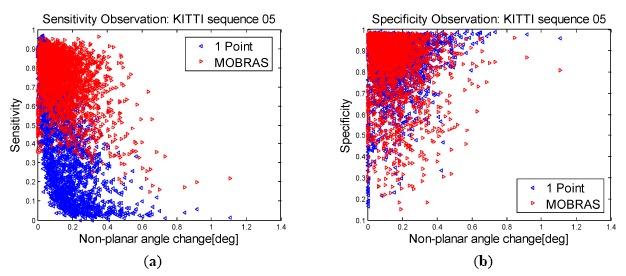
Sensitivity and Specificity. Comparison between 1-Point and MOBRAS algorithms.

The relations between sensitivity, specificity, and the non-planar-angle component of the rotation (defined as pitch angle here) are depicted in Figure 6. From the distribution of sensitivity, MOBRAS finds on average more true inliers than the 1-point algorithm, in which detection ratio decreases significantly as the non-planar angle increases. However, the specificity plot indicates that both methods suffer from high false positive rate, which can undermine the inlier quality and undermine the motion estimation result. However, we will show in the following sections that by introducing the linearized pitch angle component, motion estimation accuracy can be refined and improved.

## 4. Dynamic Model Based MYP Algorithm

In this section we explore the relationship between yaw angle increment *ω* and side slip angle *β*, and try to represent *β* as a linear combination of ω and other measurable parameters. We also present how to solve *ω* and pitch change *γ* with this parameterization. The processing time of our MYP algorithm is the same as the 1-point algorithm but it improves the estimation accuracy significantly.

### 4.1. Exploring the Relationship between Yaw Rate and Side Slip Angle

Recall that in Equations (7) and (8), we want to derive the relationship between yaw rate *ω*_r_ and side slip angle *β*, which are the principal components of vehicle motion. Because we do not need to estimate wheel steering angle *δ*, it is easy to eliminate it by multiplying Equation (7) by *l*_F_ and subtracting Equation (8) from it. The result of this elimination is:

(10) $$(l_{\text{F}}+l_{\text{R}})k_{\text{R}}\beta -\frac{l_{\text{R}}}{v_{\text{long}}}(l_{\text{F}}+l_{\text{R}})k_{\text{R}}\omega_{\text{r}}=m\cdot l_{\text{F}}\cdot {\dot{v}}_{\text{lat}}+m\cdot l_{\text{F}}\cdot v_{\text{long}}\cdot \omega_{\text{r}}-I_{y}{\dot{\omega }}_{\text{r}}$$

Or:

(11) $$\beta =l_{\text{R}}\frac{\omega_{\text{r}}}{v_{\text{long}}}+\frac{m}{k_{\text{R}}}\cdot \frac{l_{\text{F}}}{l}\cdot a_{\text{lat}}+\frac{m}{k_{\text{R}}}\cdot \frac{l_{\text{F}}}{l}\cdot v_{\text{long}}\cdot \omega_{\text{r}}-\frac{I_{y}}{l\cdot k_{\text{R}}}{\dot{\omega }}_{\text{r}}$$

where *l* = *l*_F_ + *l*_R_ denotes the wheelbase of the vehicle, and *a*_lat_ represents lateral acceleration.

As shown in Equation (11), the side slip angle *β* can be represented as a linear function of yaw rate *ω*_r_. Notice that if monocular set longitudinal velocity *v*_long_ is known (provide by other sensors), *l*_F_, *l* are geometric parameters and can be measured manually. *m, k*_R_ and *I*_y_ can be estimated off-line using an identification method.

### 4.2. Parameterization of the Motion

Similar to the model in [12], local road planar hypothesis is also tenable in this work because the moving trajectory relates to unsprung mass. MYP algorithm (1) represents the side slip angle *β* as the linear function of yaw angle change *ω*, like *β* = *c*_1_*ω* + *c*_2_, where $c_{1}=\left(\frac{l_{\text{R}}}{v_{\text{long}}}+\frac{m}{k_{\text{R}}}\cdot \frac{l_{\text{F}}}{l}\cdot v_{\text{long}}\right)/\text{d}t$, $c_{2}=\frac{m}{k_{\text{R}}}\cdot \frac{l_{\text{F}}}{l}\cdot a_{\text{lat}}-\frac{I_{y}}{l\cdot k_{\text{R}}}{\dot{\omega }}_{\text{r}}$, d*_t_* is the acquisition interval; and (2) adds the rotation component caused by pitch angle change.

First, the coefficients *c*_1_ and *c*_2_ can be calculated using known geometrical parameters *l*_F_, *l*, measured parameters *v*_long_, *a*_lat_ and off-line trained parameters m, *k*_R_ and *I*_y_ as shown in Equation (11). The off-line training method for these parameters will be described later in this section. Second, as we observed in both synthetic and real car data, we found that the pitch angle change *γ* was maintained to be less than 2° per 100 ms, it is reasonable to assume sin *γ* ≈ *γ*, cos *γ* ≈ 1, finally the essential matrix is parameterized as:

(12) $$\begin{array}{c} E=\left[\begin{array}{ccc} 0 & -1 & 0\\ 1 & 0 & -\tan\beta \\ 0 & \tan\beta & 0 \end{array}\right]\cdot \left[\begin{array}{ccc} \cos\omega & 0 & -\sin\omega \\ 0 & 1 & 0\\ \sin\omega & 0 & \cos\omega \end{array}\right]\cdot \left[\begin{array}{ccc} 1 & 0 & 0\\ 0 & 1 & -\gamma \\ 0 & \gamma & 1 \end{array}\right]\\ E=\left[\begin{array}{ccc} 0 & -1 & \gamma \\ \cos\omega -\tan\beta \sin\omega & -\gamma (\sin\omega +\cos\omega \tan\beta ) & -(\sin\omega +\cos\omega \tan\beta )\\ 0 & \tan\beta & -\gamma \tan\beta \end{array}\right] \end{array}$$

Substituted into Epipolar Constraint Equation (3), the equation constrained by feature correspondences is:

(13) $$\begin{array}{l} x_{k-1}y_{k}(\cos\omega -\tan\beta \sin\omega )-x_{k}y_{k-1}-y_{k-1}y_{k}\gamma (\sin\omega +\cos\omega \tan\beta )\\ +y_{k-1}\tan\beta +x_{k}\gamma -y_{k}(\sin\omega +\cos\omega \tan\beta )-\gamma \tan\beta =0 \end{array}$$

where *x_k_*_−1_, *y_k_*_−1_, *x_k_, y_k_* are elements of calibrated image coordinates ***q̂***_*k*−1_ = [*x_k_*_−1_ *y_k_*_−1_ 1]*^T^* and ***q̂***_*k*_ = [*x_k_* *y_k_*1]*^T^* of a couple of feature points.

Equation (13) contains complex trigonometric polynomials and it is difficult to solve for *ω*. Practically, in both synthetic data and real data, the yaw angle increment are typically less than 10° (per 100 ms). We use Taylor expansion of trigonometric functions and neglect the term with orders higher than O(*ω*^2^).

(14) $$\begin{array}{l} \begin{array}{ll} \sin\omega \approx \omega & \cos\omega \approx 1-\frac{\omega^{2}}{2} \end{array}\\ \tan\beta =\tan(c_{1}\omega +c_{2})\approx c_{1}\omega +c_{2}+\frac{{\left(c_{1}\omega +c_{2}\right)}^{3}}{3}\approx c_{2}+\frac{c_{2}^{3}}{3}+c_{1}(1+c_{2}^{2})\omega +c_{1}^{2}c_{2}\omega^{2} \end{array}$$

Combing the simplification in Equations (13) and (14), we obtain a quadratic of *ω* and *γ*, which can be solved using Root Formula, Sturm-sequences, or any other root-solve algorithm realized by current library, like Eigen [25]. After that, *β* can be also recovered. Obviously, at most, two solutions will be obtained, which is much less than 11 possible solutions of the 5-point algorithm.

### 4.3. Off-Line Parameters Identification

As mentioned before, we need to estimate parameters including *m, k*_R_ and *I*_y_ using an optimization approach. An error function is defined as the sum of all normalized error between modeled outputs and measured values. Let *β˜_k_* denote side slip angle measured in frame *k* and define coefficient matrix *A* as:

(15) $$\begin{array}{l} \text{A}(k,1)=l\cdot {\tilde{\beta }}_{i}-l_{\text{R}}\cdot {\dot{\omega }}_{\text{r}}(k)/v_{\text{long}}(k)\\ \text{A}(k,2)=-l_{\text{F}}\cdot \left({\dot{v}}_{\text{lat}}(k)+v_{\text{long}}(k)\cdot {\dot{\omega }}_{\text{r}}(k)\right)\\ \text{A}(k,3)={\dot{v}}_{\text{lat}}(k) \end{array}$$

Equation (15) is overdetermined and *m/k*_R_, *I*_y_/*k*_R_ can be solved in least square sense. We only take longitude velocity as input, and other measure parameters including lateral velocity, yaw rate, longitude acceleration, lateral acceleration are calculated using the estimation results from the previous frame.

It is noteworthy that in practice only the ratios between m and *k*_R_ and *I*_y_ and *k*_R_ are needed, rather than their individual values. Using only KITTI Sequence 01 for training, we obtained *m/k*_R_ = 0.0073, *I*_y_/*k*_R_ = 0.0084. Empirically we assume that the vehicle mass equals to 1500 kg and the rear tire cornering stiffness is *k*_R_ ≈ 205,480 N/rad, the moment of inertia around the *y*-axis is *I*_y_ ≈ 1726 kg·m^2^. The trained parameters obtained here are also used in the benchmark tests in Section 6.3.

**Figure 7.**
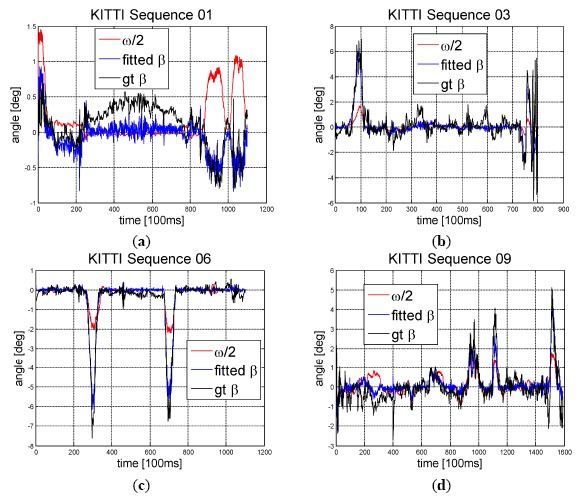
Fitted side slip angle using trained parameters: gt denotes ground truth.

The identification and test results are shown in Figure 7. All plots of Sequence 03, 06 and 09 demonstrate that when big turning occurred, side slip angle *β* calculated using the dynamic model is more accurate than *ω*/2, which is derived using only Ackerman Steering Principle. This characteristic will directly affect the estimation accuracy of the algorithm in sharp turning scenario.

### 4.4. Inliers Segmentation

As mentioned in Section 2.1, the model estimation *R*_1_ and *t*_1_ can be used to select inliers through thresholding the reprojection errors. Since MYP is essentially a model of 2 DoF, which requires two feature correspondences to compute a motion hypothesis. It can be implemented in either RANSAC scheme or a two-dimensional histogram. In this work we use the latter considering the deterministic of estimation results. The implementation of MYP can be concluded below.

(1) For feature correspondence {*^i^****q****_k_*_−1_↔*^i^****q****_k_*}, {*^j^****q****_k_*_−1_↔*^j^****q****_k_*}, *i* = 1:*N* − 1, *j* = *I* + 1:*N*, solve *^ij^ω* and *^ij^γ* using Equations (13) and (14) couple wise.
(2) Conduct 2D histogram with specific resolution and range, vote with *^ij^ω* and *^ij^γ*.
(3) Select the winner model estimation {ω*, γ*} = argmax{histogram}.
(4) Calculate β* according to Equation (11), as well as model estimation rotation matrix and translation vector:
  (16) $${\text{R}}^{\text{m}}=\left[\begin{array}{ccc} \cos\omega^{*} & 0 & -\sin\omega^{*}\\ 0 & 1 & 0\\ \sin\omega^{*} & 0 & \cos\omega^{*} \end{array}\right]\cdot \left[\begin{array}{ccc} 1 & 0 & 0\\ 0 & \cos\gamma^{*} & -\sin\gamma^{*}\\ 0 & \sin\gamma & \cos\gamma^{*} \end{array}\right]\;{\text{t}}^{\text{m}}=\left[\begin{array}{c} \sin\beta^{*}\\ 0\\ \cos\beta^{*} \end{array}\right]$$
(5) Triangulate {*^i^****q****_k_*_−1_↔*^i^****q****_k_*} with *R*_m_ and *t*_m_ to obtain 3D estimation point {*^i^**Q̂***}, then calculate reprojection error and distinguish inliers and outliers with some threshold.

## 5. Refinement

As a hypothesis generator in relative pose estimation based on hypothesis-test-validation framework, minimal set solving algorithms are not able to provide accurate solution because error exists in the coordinates of point correspondences. Consequently, all inliers that have been found should be used to obtain an accurate solution in the least square sense.

However, the winner of minimal solutions is not far from the true solution. It can work as a good starting point in some iterative non-linear algorithms for solving the minimization problem. In most optimization-based relative pose recovery algorithms, nine elements in the rotation matrix *R* are treated as independent variables, and the orthogonality of *R* cannot be preserved iteratively due to this inherent deficiency that rotation matrix has no explicit geometrical meaning. In monocular VO problem we can use three attitude angles as optimization variables, then the orthogonality problem will be natively raveled out. The computational efficiency of Euler angle parameter optimization seems low due to the trigonometric functions existing in the representation of *R*. Many efficient extreme search methods like conjugate gradient method, variable metric methods, and Levenberg-Marquardt method, *etc*., can work well, given explicit gradient expression of objective function. Then the cost function and its gradient function based on Euler angle parameterized motion expressions can be derived. Here, we refine *R* and *t* in turns, the method that compelled decoupling of *R* and *t* was also used by [26], and obtained promising result in this research.

## 6. Experiments

In this section, we compare our MYP algorithm with 1-point, MOBRAS and standard 5-point algorithm on both synthetic data and real car benchmark data.

### 6.1. CarSim Based Simulation Tests

First the inlier identification performance of our MYP algorithm was investigated with synthetic data generated by the joint simulation platform conducted using CarSim and Matlab in our previous work [23]. Real-time vehicle status parameters including velocity, pitch, roll and yaw angles were generated by CarSim [27]. Meanwhile, the positions were also recorded and used as ground truth.

In order to make the simulation more realistic, we set the simulation parameters as shown in Table 1. We varied the outlier percentage between 10% and 70% to compare the performance of mentioned algorithms in different levels of outliers.

We compared our MYP algorithm with the 1-point algorithm [12], MOBRAS algorithm [15], and standard 5-point RANSAC [17] using the generated synthetic data. We used 500 random iterations in the 5-point RANSAC method, which, according to the RANSAC statistics [1], should provide a probability of success of 99.42% (calculated assuming a fraction of outliers equal to 60%). We used the recommended value in [15] for the sampling number of MORRAS.

**Figure 8.**
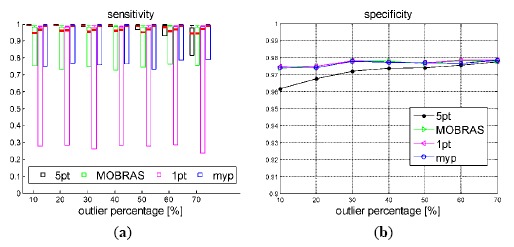
Sensitivity and specificity of true inliers detection *vs.* outlier percentage. Vehicle velocity is fixed at 50 km/h.

**Figure 9.**
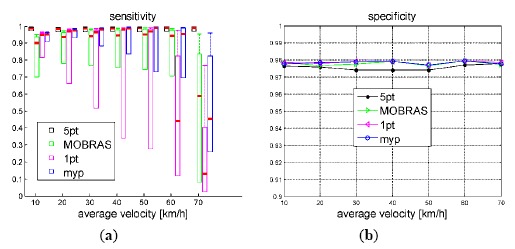
Sensitivity and specificity of true inliers detection *vs.* vehicle velocity. Outlier percentage is set to 50%.

As defined in Section 3.3, sensitivity and specificity were used to evaluate the inlier identification performance. The resulting statistics are shown in Figures 8 and 9. Thick red bars denote the median of the errors. The higher border of the rectangles denote 75% percentiles while the lower borders represent the minimum value of the errors; the top end of the dash lines denotes 90% percentiles.

As Figure 8 illustrates, the inlier detection performance of motion model based methods (1-point, MOBRAS and MYP) were not affected by the percentage of outliers, but the detect ratio of 5-point algorithm decreased as the outlier percentage increased. It is determined by its nature that 5-point RANSAC is a probabilistic method. Whereas, model based methods grasp the main components of vehicle motion better. However, 5-point algorithm was the most robust method to the vehicle velocity when the outlier percentage was below 50%. Meanwhile the 1-point and MOBRAS algorithms were quite vulnerable to velocity level. They only worked well when the velocity was neither too low nor too high. Their performance confirmed that the ratio *β/ω* = 1/2 fits well when the vehicle moves at mid velocity around 50 km/h as shown in Figure 3.

**Figure 10.**
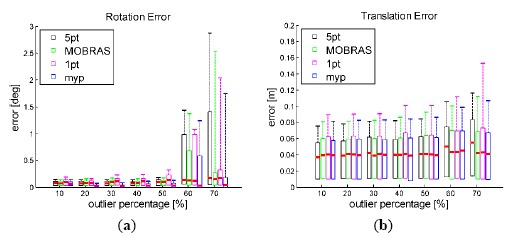
Accuracy of final motion estimation *vs.* outlier percentage. Vehicle velocity is fixed at 50 km/h.

**Figure 11.**
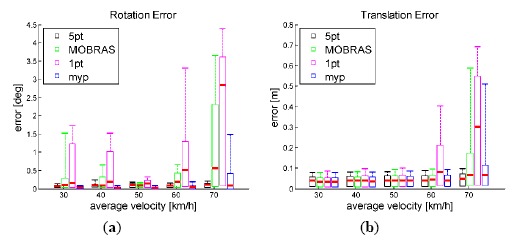
Accuracy of final motion estimation vehicle velocity level. Outlier percentage is set to 50%.

Figures 10 and 11 show the accuracy of different inlier detection methods combing with the same final refinement. It is noteworthy that the absolute scale *s* of the translation vector is calculated by velocity *vel* and time interval ΔT as:

(17) s=vel·ΔT

where ΔT equals to 0.1 s in the simulation as well as in real car test on the KITTI dataset.

The accuracy performance is consistent with the Sensitivity illustrated in Figures 8 and 9. Vehicle velocity variation did not affect the accuracy of standard 5-point RANSAC. When the vehicle velocity was around 50 km/h, 1-point algorithm as well as MORBAS that are based on kinematic models obtained the highest accuracy and the median values of their rotation errors exhibited a U-shaped distribution. The accuracy of MYP was slightly lower than 5-point RANSAC in high speed situations. On the other hand, when the percentage of outliers increased, 5-point RANSAC suffered the most while the motion model-based algorithms exhibited more robustness, wherein MYP obtained the best performance.

Table 2 shows the comparison of execution time among four monocular algorithms. The most efficient method was the 1-point algorithm, which on average took less than 2 ms. MOBRAS was more than 50 times slower than the 1-point algorithm because internally MOBRAS requires 100 1-point samplings. In our experiments, the 5-point RANSAC ran even slower than MOBRAS, around 23 times. This is consistent with the results presented in [25] which claimed that the 5-point RANSAC is 50 times slower than MOBRAS. Our MYP ran 10 times faster than MOBRAS and 240 times faster than the 5-point RANSAC. Referring to the accuracy performance shown in Figures 10 and 11, MYP algorithm had the highest performance over cost ratio.

### 6.2. Influence of Accuracy of the Velocity

We investigated the influence of the velocity accuracy on the performance of the proposed algorithm, since one of the inputs of MYP algorithm is the vehicle velocity. Using the same simulation platform as Section 6.1, different levels of velocity error were randomly generated and added to the input of the MYP algorithm.

**Figure 12.**
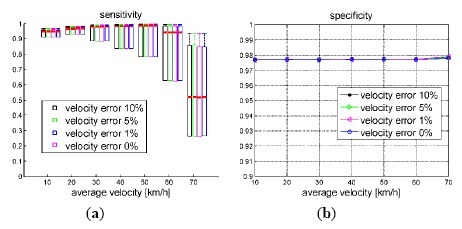
Sensitivity and specificity of true inliers detection *vs.* vehicle velocity. Outlier percentage is set to 50%, velocity error varies from 0 to 10%.

**Figure 13.**
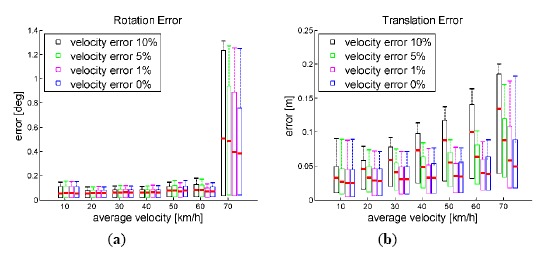
Rotation and translation error *vs.* vehicle velocity. Outlier percentage is set to 50%, velocity error varies from 0 to 10%.

As Figure 12 shows, the inlier detection performance of MYP was almost not affected by the velocity error (up to 10%). But according to Figure 13, the rotation error of MYP slightly increased as the velocity error increased. On the other hand, the translation error grew more significantly. It is true because the scale of translation is completely determined by velocity.

### 6.3. Test on Benchmark Data

We evaluated our MYP algorithm on the KITTI dataset, which consists of 22 video sequences. The raw image resolution of these videos is 1392 × 512 pixels, and the frame rate is on average 10 frames per second. Ground truth is provided for training Sequences 00–10. There is no ground truth for test Sequences 11–21. However, our MYP algorithm like other monocular methods requires the input of vehicle velocity. Hence we only ran on left images of Sequences 00–10 for training and the vehicle velocity was calculated using distances between positions.

We compared accuracy over Sequences 00–10. An overview of the average translation and rotation errors calculated over the eleven test sequences for three different algorithms are shown in Table 3. The results are sorted according to the average rotation error. According to this evaluation metrics, our MYP outperformed other methods.

In order to make the experiments more realistic, we also tested MYP algorithm with different velocity error on the same datasets. The results are shown in Table 4. MYP outperformed 5-point RANSAC even when velocity is measured with 5% error; MYP obtained almost the same performance as MOBRAS when velocity was error up to 10%.

**Figure 14.**
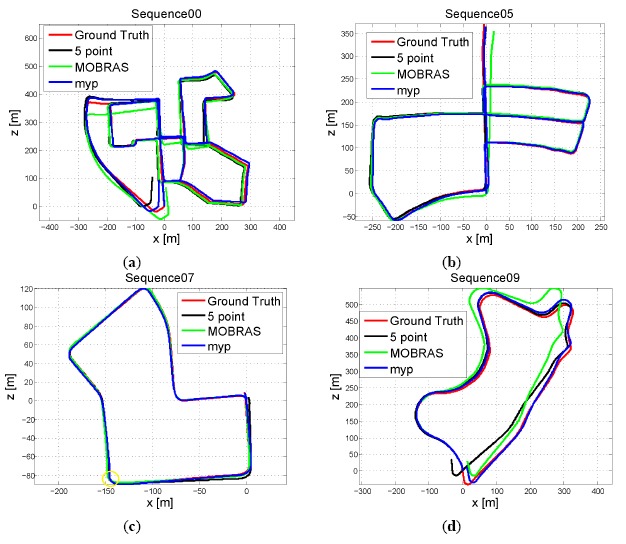
Exemplary estimated trajectories: Comparison between MYP, MOBRAS and 5-point RANSAC.

**Figure 15.**
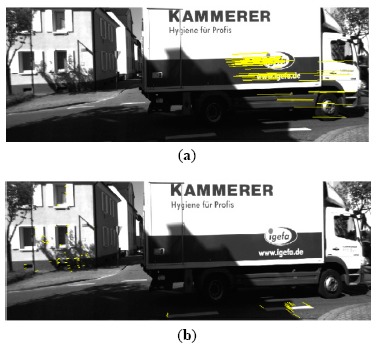
Moving obstacle causes complex situation with high outlier percentage. Yellow lines denote false feature correspondences. (**a**) Inliers preserved by 5-point RANSAC; (**b**) Inliers preserved by MYP.

A few exemplary estimated trajectories are shown in Figure 14. Of the three methods compared, MYP suffered the smallest drift. This is due to that the rotation error is the main source of drift and MYP has the smallest rotation estimation error as shown in Table 3. Another interesting finding was that both MYP and MOBRAS outperformed the 5-point RANSAC algorithm in Sequence 07. This was because in a complex scenario (marked by the yellow circle), high percentage of outliers caused by crossing vehicles undermined the estimation of the 5-point RANSAC algorithm. Figure 15 shows an example of a crossing vehicle as a moving obstacle that causes high outlier percentage and affects the performance of the 5-point RANSAC algorithm.

The execution time of our MYP algorithm pipeline including feature detection and tracking was around 60 ms. Our test platform used a PC with an Intel(R) Core(TM)2 CPU Q9500 at 2.83 GHz and 4 GB of RAM. The experimental results showed that our algorithm was capable of operating at the frame rate of 15 Hz, with the maximum execution times well below 100 ms. Our algorithm performed several times faster than other algorithms while maintaining better or at least comparable accuracy. Such a low latency system is well suited for real-time autonomous vehicles.

## 7. Conclusions

In this paper, we have presented a novel and fast visual odometry algorithm called MYP for wheeled vehicles moving in dynamic outdoor environments. The core of MYP is single track bicycle dynamic model and reasonable linear approximation based 2-DoF inlier selection method, which captures three most important motion components, namely yaw angle increment, side slip angle and pitch angle increment. Furthermore, MYP is fully deterministic compared to random sampling based RANSAC VO methods.

We successfully tested our algorithm on a large image dataset, spanning dozens of kilometers. Experiments showed that our MYP algorithm outperformed state-of-the-art monocular visual odometry approaches in terms of accuracy and efficiency. We also demonstrated that MYP can handle a variety of different types of moving obstacles, achieve accurate position and orientation estimations, and meet the localization and navigation requirements of intelligent vehicles in complex urban environments.

However, there is a limitation of MYP' application in higher speed situations. In the single track bicycle dynamic model, the tire stiffness characteristics exhibit good linearity. In this case, MYP can get good side slip angle fitting results when the vehicle velocity is not too high. In high-speed scenarios, typically higher than 70 km/h, the tire stiffness characteristics exhibit nonlinearity, which leads to performance degradation of the MYP algorithm. In our future work, we will extend MYP to high speed driving conditions by considering nonlinear vehicle dynamic models.

## Figures and Tables

**Table 1.** Simulation parameters.

**Parameters**	**Value**
Simulation time step	100 [ms]
Expectation velocity	30:10:70 [km/h]
Outlier percentage	10:10:70 [%]
Std of gaussian noise add to image points σ	0.5 [pixel]
Reprojection error threshold	1.0 [pixel]
Iteration number of 5-point alogrithm	500
Sampling number of MOBRAS	100

**Table 2.** Execution time of outlier rejection process.

**Algorithm**	**Mean [ms]**	**Median [ms]**	**Min [ms]**	**Max [ms]**
5-point RANSAC	2106.3	1936.9	455	3513.8
MOBRAS	90	91	61	108
1-point algorithm	1.9	1.8	1.5	2.9
MYP	8.6	8.7	6.4	10.6

**Table 3.** Overview of average translation and rotation error on KITTI Sequences 00–10.

**Method**	**Rotation**	**Translation**
MYP	0.0145 [deg/m]	1.32%
5-Point RANSAC	0.0161 [deg/m]	1.36%
MOBRAS	0.0172 [deg/m]	1.39%

**Table 4.** Average performance of MYP against velocity error.

**Velocity Error**	**Rotation**	**Translation**
0%	0.0145 [deg/m]	1.32%
5%	0.0156 [deg/m]	1.45%
10%	0.0173 [deg/m]	1.65%
